# Supplementary material for: Detection of spreader nodes in human-SARS-CoV protein-protein interaction network
Source: PeerJ. 2021 Sep 6;9:e12117. doi: 10.7717/peerj.12117 (PMC8428263; doi:10.7717/peerj.12117)
Supplement: Supplemental Information 1 [file peerj-09-12117-s001.pdf]

Detection of spreader nodes in Human-SARS-CoV protein-protein interaction network: Supplementary Document

1. Algorithm of Synthetic PPIN formation

Algorithm 1: Synthetic PPIN Formation

Input: PPIN represented by an undirected graph where each vertex represents a protein and edge represents the interactions.
No. of nodes (k)

Output: Synthetic PPIN with given no. of nodes (k)

```
Begin
//formation of a list of unique nodes
  M = ∅//Let M be an empty list
  k = 0
// i ≠ j and 1<=i, j<=n, n is the total no. of proteins in PPIN
  for each combination of protein pair (Pi,Pj) in PPIN
      split the protein pair (Pi,Pj)
      append each protein Pi and Pj to M
      k = k + 1
   end for
//Formation of unique set of proteins by applying set ()
  set M=list(set(M))
// Random selection of nodes by importing random module and storing in random_nodes
  set random_nodes to random.sample(nodes,k)
//Display selected random nodes
  output (random_nodes)
//Display of interactions of selected random nodes i.e., synthetic PPIN
  for each protein Pi in random_nodes
      for each combination of protein pair (Pi,Pj) in PPIN
         if protein Pi present in protein pair (Pi,Pj)
             output (protein pair (Pi,Pj))
         end if
      end for
  end for
End
```

2. Studied Models in epidemiology

Compartmental models are a very general modelling technique. They are often applied to the mathematical modelling of infectious diseases. The ***SIR model*** is one of the simplest compartmental models, and many models are derivatives of this basic form. The model consists of three compartments: -

S: The number of susceptible individuals. When a susceptible and an infectious individual come into "infectious contact", the susceptible individual contracts the disease and transitions to the infectious compartment.

I: The number of infectious individuals. These are individuals who have been infected and are capable of infecting susceptible individuals.

R: The number of removed (and immune) or deceased individuals. These individuals have been infected and have either completely recovered from the disease and entered the removed compartment (completely immune to the disease), or died. It is assumed that the number of deaths is negligible concerning the total population. This compartment may also be called "recovered" or "resistant". They will not be again infected with the same disease.

Some infections, for example, those from the common cold and influenza, do not confer any long-lasting immunity. Such infections do not give immunity upon recovery from infection, and individuals become susceptible again. So, for these kinds of infections ***SIS model*** is used. The model consists of three compartments: -

S: The number of susceptible individuals. When a susceptible and an infectious individual come into "infectious contact", the susceptible individual contracts the disease and transitions to the infectious compartment.

I: The number of infectious individuals. These are individuals who have been infected and are capable of infecting susceptible individuals.

S: Individuals get recovered and again become susceptible.

Out of SIR (susceptible, infected, and recovered) and SIS model (susceptible, infected, and susceptible), SIS is the standard one because from the viral infection perspective, there is nothing called a completely recovered state (**R**) because if someone gets infected with viral disease then they will avail drugs for the disease and will get cured. But they again become susceptible to the same disease (**S**). Therefore, it cannot be guaranteed that the viral disease will not happen again after getting cured. So, the SIS model is given more priority than SIR in this proposed work.

3. Supplementary Tables

Table S1 Ranked spreader edges between SARS-CoV s level 1 and level 2 human spreaders at high threshold

	**Spreader Edges**		
**Rank**	**SARS-CoV s level 1 and level 2 human spreaders**		**Spreading ability of spreader edges**
1	APP	UBE2I	9297903
2	APP	CAV1	8882769
3	APP	PPIA	8743925
4	APP	EIF3F	8737730
5	BCL2A1	APP	8640455
6	ELAVL1	EEF1A1	4284371

7	NCL	ELAVL1	4174604
8	PPIA	ELAVL1	4168684
9	BCL2L2	ELAVL1	4065937
10	NXF1	UBE2I	3653905
11	NXF1	SGTA	3402603
12	NTRK1	YWHAE	3275095
13	NXF1	CAV1	3238771
14	NXF1	EEF1A1	3215615
15	XPO1	UBE2I	3169805
16	DCTN2	NXF1	3066498
17	NTRK1	CAV1	3038977
18	NXF1	MCL1	3038159
19	NTRK1	EEF1A1	3015820
20	XPO1	YWHAE	2990790
21	NCL	NTRK1	2906052
22	NTRK1	PPIA	2900133
23	NTRK1	EIF3F	2893938
24	DCTN2	NTRK1	2866704
25	GRB2	UBE2I	2349446
26	GRB2	YWHAE	2170431
27	IKBKB	MEOX2	2061694
28	UBC	SGTA	2048943
29	YWHAE	EGFR	2023580
30	GRB2	PFDN5	1943610
31	GRB2	EEF1A1	1911156

Table S2 Ranked spreader edges between SARS-CoV s level 1 and level 2 human proteins at medium threshold

	**Spreader Edges**		
**Rank**	**SARS-CoV s level 1 and level 2 human spreaders**		**Spreading ability of spreader edges**
1	APP	UBE2I	9297902.664
2	APP	CAV1	8882768.796
3	APP	PPIA	8743924.63
4	APP	EIF3F	8737729.873
5	BCL2A1	APP	8640455.016
6	ELAVL1	EEF1A1	4284371.281
7	NCL	ELAVL1	4174603.596
8	PPIA	ELAVL1	4168683.646
9	BCL2L2	ELAVL1	4065936.721
10	NXF1	UBE2I	3653905.316
11	NXF1	SGTA	3402602.703
12	NTRK1	YWHAE	3275094.903

13	NXF1	CAV1	3238771.448
14	NXF1	EEF1A1	3215614.916
15	XPO1	UBE2I	3169805.311
16	DCTN2	NXF1	3066498.385
17	NTRK1	CAV1	3038976.693
18	NXF1	MCL1	3038159.191
19	NTRK1	EEF1A1	3015820.161
20	XPO1	YWHAE	2990789.654
21	NCL	NTRK1	2906052.476
22	NTRK1	PPIA	2900132.526
23	NTRK1	EIF3F	2893937.769
24	DCTN2	NTRK1	2866703.629
25	GRB2	UBE2I	2349446.25
26	GRB2	YWHAE	2170430.592
27	IKBKB	MEOX2	2061694.24
28	UBC	SGTA	2048942.842
29	YWHAE	EGFR	2023579.783
30	GRB2	PFDN5	1943609.51
31	GRB2	EEF1A1	1911155.85
32	UBC	EEF1A1	1861955.055
33	TP53	UBE2I	1829375.898
34	BAG3	UBE2I	1809920.855
35	NCL	GRB2	1801388.165
36	GRB2	PPIA	1795468.215
37	CAV1	EGFR	1787461.572
38	GOLGA2	UBE2I	1779690.372
39	EEF1A1	EGFR	1764305.041
40	DCTN2	GRB2	1762039.318
41	NCL	UBC	1752187.37
42	EIF3F	UBC	1740072.663
43	YWHAE	SHMT2	1738748.567
44	DCTN2	UBC	1712838.523
45	BCL2A1	GRB2	1691998.601
46	MCL1	UBC	1684499.329
47	TP53	YWHAE	1650360.24
48	EIF3F	EGFR	1642422.649
49	PROP1	UBE2I	1641256.177
50	BAG3	YWHAE	1630905.197
51	CDK2	UBE2I	1622979.319
52	DCTN2	EGFR	1615188.509
53	CUL3	YWHAE	1614438.165
54	TRIM27	UBE2I	1579133.993
55	CEACAM1	EGFR	1537728.445
56	HSP90AA1	YWHAE	1503676.376

57	TRAF6	UBE2I	1451472.666
58	CDK2	YWHAE	1443963.661
59	MCM2	YWHAE	1440253.354
60	BRCA1	UBE2I	1439839.399
61	HSP90AA1	SGTA	1431389.421
62	ESR1	UBE2I	1424801.897

Table S3 Ranked spreader edges between SARS-CoV s level 1 and level 2 human spreaders at low threshold

	**Spreader Edges**		
**Rank**	**SARS-CoV s level 1 and level 2 human spreaders**		**Spreading ability of spreader edges**
1	APP	UBE2I	9297902.664
2	APP	CAV1	8882768.796
3	APP	PPIA	8743924.63
4	APP	EIF3F	8737729.873
5	BCL2A1	APP	8640455.016
6	ELAVL1	EEF1A1	4284371.281
7	NCL	ELAVL1	4174603.596
8	PPIA	ELAVL1	4168683.646
9	BCL2L2	ELAVL1	4065936.721
10	NXF1	UBE2I	3653905.316
11	NXF1	SGTA	3402602.703
12	NTRK1	YWHAE	3275094.903
13	NXF1	CAV1	3238771.448
14	NXF1	EEF1A1	3215614.916
15	XPO1	UBE2I	3169805.311
16	DCTN2	NXF1	3066498.385
17	NTRK1	CAV1	3038976.693
18	NXF1	MCL1	3038159.191
19	NTRK1	EEF1A1	3015820.161
20	XPO1	YWHAE	2990789.654
21	NCL	NTRK1	2906052.476
22	NTRK1	PPIA	2900132.526
23	NTRK1	EIF3F	2893937.769
24	DCTN2	NTRK1	2866703.629
25	GRB2	UBE2I	2349446.25
26	GRB2	YWHAE	2170430.592
27	IKBKB	MEOX2	2061694.24
28	UBC	SGTA	2048942.842
29	YWHAE	EGFR	2023579.783
30	GRB2	PFDN5	1943609.51
31	GRB2	EEF1A1	1911155.85

32	UBC	EEF1A1	1861955.055
33	TP53	UBE2I	1829375.898
34	BAG3	UBE2I	1809920.855
35	NCL	GRB2	1801388.165
36	GRB2	PPIA	1795468.215
37	CAV1	EGFR	1787461.572
38	GOLGA2	UBE2I	1779690.372
39	EEF1A1	EGFR	1764305.041
40	DCTN2	GRB2	1762039.318
41	NCL	UBC	1752187.37
42	EIF3F	UBC	1740072.663
43	YWHAE	SHMT2	1738748.567
44	DCTN2	UBC	1712838.523
45	BCL2A1	GRB2	1691998.601
46	MCL1	UBC	1684499.329
47	TP53	YWHAE	1650360.24
48	EIF3F	EGFR	1642422.649
49	PROP1	UBE2I	1641256.177
50	BAG3	YWHAE	1630905.197
51	CDK2	UBE2I	1622979.319
52	DCTN2	EGFR	1615188.509
53	CUL3	YWHAE	1614438.165
54	TRIM27	UBE2I	1579133.993
55	CEACAM1	EGFR	1537728.445
56	HSP90AA1	YWHAE	1503676.376
57	TRAF6	UBE2I	1451472.666
58	CDK2	YWHAE	1443963.661
59	MCM2	YWHAE	1440253.354
60	BRCA1	UBE2I	1439839.399
61	HSP90AA1	SGTA	1431389.421
62	ESR1	UBE2I	1424801.897
63	COPS5	UBE2I	1402029.245
64	TP53	EEF1A1	1391085.498
65	CAV1	MOV10	1381399.319
66	ZDHHC17	EEF1A1	1380592.242
67	UBE2I	ATXN1	1362088.512
68	MOV10	EEF1A1	1358242.787
69	TMEM17	CAV1	1356149.641
70	CUL3	EEF1A1	1355163.423
71	HSPB1	UBE2I	1341884.753
72	MYC	YWHAE	1341253.761
73	LNX1	PFDN5	1333010.914
74	DCTN2	SHMT2	1330357.294
75	TP53	IKBKB	1314401.976

76	YWHAE	FBXO6	1313852.619
77	HGS	UBE2I	1294714.22
78	TP53	NCL	1281317.814
79	CUL3	IKBKB	1278479.9
80	YWHAE	VCP	1277472.997
81	TRAF2	YWHAE	1274995.25
82	TP53	BCL2	1268975.057
83	HSP90AA1	CAV1	1267558.165
84	BAG3	NCL	1261862.77
85	MDM2	UBE2I	1260258.833
86	YWHAZ	YWHAE	1255600.341
87	BAG3	BCL2	1249520.013
88	NCL	MOV10	1248475.102
89	CUL3	NCL	1245395.738
90	HSP90AA1	EEF1A1	1244401.634
91	CUL3	PPIA	1239475.788
92	TP53	BCL2L1	1239080.263
93	EWSR1	PPIA	1229282.205
94	FN1	YWHAE	1214083.458
95	TP53	MCL1	1213629.773
96	EP300	UBE2I	1213483.256
97	CUL3	DCTN2	1206046.891
98	CDK1	UBE2I	1205680.466
99	UBE2I	AGTRAP	1198756.302
100	HDAC1	UBE2I	1194236.516
101	BAG3	MCL1	1194174.73
102	IKBKE	UBE2I	1186760.92
103	LNX1	PPIA	1184869.618
104	CDK2	EEF1A1	1184688.919
105	YWHAE	ATXN1	1183072.854
106	MCM2	EEF1A1	1180978.612
107	PFDN5	SDCBP	1178978.137
108	TCF4	UBE2I	1174611.299
109	TP53	BCL2L2	1172650.939
110	HSP90AA1	IKBKB	1167718.111
111	CSNK2B	UBE2I	1166588.488
112	HSPB1	YWHAE	1162869.095
113	JUN	UBE2I	1162163.465
114	KDM1A	UBE2I	1155214.334
115	CDC37	UBE2I	1151160.054
116	TRIM23	UBE2I	1145591.625
117	HSP90AA1	NCL	1134633.949
118	HSP90AA1	BCL2	1122291.192
119	BMI1	UBE2I	1120564.971

120	CCDC8	YWHAE	1118799.628
121	CSNK2A1	CAV1	1117578.493
122	MYC	PFDN5	1114432.68
123	HSF2BP	UBE2I	1113581.235
124	FBXW11	YWHAE	1086642.037
125	SRC	YWHAE	1085833.441
126	NCL	CDK2	1074921.234
127	NCL	MCM2	1071210.927
128	PPIA	CDK2	1069001.284
129	CALM1	YWHAE	1067438.977
130	MCM2	PPIA	1065290.977
131	IKBKB	TRIM27	1064160.071
132	BCL2	CDK2	1062578.478
133	MCM2	EIF3F	1059096.22
134	TERF2	UBE2I	1055418.427
135	FBXO6	EEF1A1	1054577.877
136	UNC119	UBE2I	1052917.816
137	CAV1	VCP	1041354.787
138	BAG6	SGTA	1039029.262
139	TRAF2	CAV1	1038877.04
140	YWHAB	YWHAE	1038503.438
141	TRAF6	CAV1	1036338.798
142	CFTR	UBE2I	1035883.116
143	YWHAQ	YWHAE	1034131.766
144	AURKA	UBE2I	1032671.376
145	CLTC	UBE2I	1030687.678
146	KAT5	UBE2I	1015856.627
147	UBE2I	TSNAX	1010628.678
148	CUL7	YWHAE	1010074.36
149	ESR1	CAV1	1009668.029
150	WNK1	UBE2I	1009409.677
151	MCL1	CDK2	1007233.194
152	CREBBP	UBE2I	1001371.822
153	HDAC5	UBE2I	999897.9437
154	FOS	UBE2I	998888.38
155	UBE2I	SIAH1	998794.2497
156	WDYHV1	PFDN5	998338.5732
157	YWHAZ	EEF1A1	996325.5986
158	UBE2I	TSG101	994223.1527
159	HNRNPA1	UBE2I	994154.0026
160	UBE2I	LMNA	993727.7467
161	BTRC	YWHAE	993713.5665
162	SH3KBP1	UBE2I	992752.976
163	TRIP13	UBE2I	992337.1069

164	NCL	CSNK2A1	984654.2768
165	YWHAG	YWHAE	983125.5399
166	CUL1	YWHAE	983107.805
167	UBE2I	SUMO2	982210.8384
168	PTEN	UBE2I	981258.9481
169	TRIM28	UBE2I	979151.0849
170	SLX4	UBE2I	976097.5171
171	APC	YWHAE	975907.5724
172	UNK	YWHAE	973611.9526
173	CSNK2A1	EIF3F	972539.5696
174	MYC	NCL	972211.3346
175	CDC37	YWHAE	972144.3969
176	MYH9	UBE2I	970752.1564
177	HLA-B	SGTA	968992.1249
178	CAV1	PRKACA	968598.7454
179	AR	UBE2I	967692.3425
180	ATF2	UBE2I	966712.2472
181	YWHAB	SGTA	966216.483
182	SUMO1	UBE2I	965376.1591
183	COPS5	EEF1A1	963738.8454
184	AKT1	YWHAE	963671.5308
185	ABL1	YWHAE	962733.2864
186	MYC	BCL2	959868.5778
187	UBE2I	SMAD4	959192.5665
188	OBSL1	YWHAE	957100.0055
189	FN1	EEF1A1	954808.7158
190	FHL3	UBE2I	954696.496
191	DAXX	UBE2I	953684.4234
192	HDAC4	UBE2I	950274.9524
193	FUS	UBE2I	949535.1125
194	SUZ12	UBE2I	948330.2296
195	TRIP6	SGTA	946975.115
196	PRKDC	UBE2I	946021.6212
197	UBE2I	UBE2D2	945725.2466
198	STAT1	UBE2I	944739.8548
199	UBE2I	TRIM54	944513.0565
200	SIRT1	UBE2I	943590.5083
201	HSPA8	YWHAE	943349.4099
202	PRKAA2	UBE2I	939213.0138
203	TRAF2	IKBKB	939036.9856
